# Inborn errors of enzymes in glutamate metabolism

**DOI:** 10.1002/jimd.12180

**Published:** 2019-10-11

**Authors:** Lynne Rumping, Esmee Vringer, Roderick H. J. Houwen, Peter M. van Hasselt, Judith J. M. Jans, Nanda M. Verhoeven‐Duif

**Affiliations:** ^1^ Department of Genetics University Medical Center Utrecht, Utrecht University Utrecht the Netherlands; ^2^ Center for Molecular Medicine University Medical Center Utrecht, Utrecht University Utrecht the Netherlands; ^3^ Department of Pediatrics University Medical Center Utrecht, Utrecht University Utrecht the Netherlands

**Keywords:** biochemical parallels, inborn errors of glutamate metabolism, newly identified IEM, phenotypic parallels

## Abstract

Glutamate is involved in a variety of metabolic pathways. We reviewed the literature on genetic defects of enzymes that directly metabolise glutamate, leading to inborn errors of glutamate metabolism. Seventeen genetic defects of glutamate metabolising enzymes have been reported, of which three were only recently identified. These 17 defects affect the inter‐conversion of glutamine and glutamate, amino acid metabolism, ammonia detoxification, and glutathione metabolism. We provide an overview of the clinical and biochemical phenotypes of these rare defects in an effort to ease their recognition. By categorising these by biochemical pathway, we aim to create insight into the contributing role of deviant glutamate and glutamine levels to the pathophysiology. For those disorders involving the inter‐conversion of glutamine and glutamate, these deviant levels are postulated to play a pivotal pathophysiologic role. For the other IEM however—with the exception of urea cycle defects—abnormal glutamate and glutamine concentrations were rarely reported. To create insight into the clinical consequences of disturbed glutamate metabolism—rather than individual glutamate and glutamine levels—the prevalence of phenotypic abnormalities within the 17 IEM was compared to their prevalence within all Mendelian disorders and subsequently all disorders with metabolic abnormalities notated in the Human Phenotype Ontology (HPO) database. For this, a hierarchical database of all phenotypic abnormalities of the 17 defects in glutamate metabolism based on HPO was created. A neurologic phenotypic spectrum of developmental delay, ataxia, seizures, and hypotonia are common in the inborn errors of enzymes in glutamate metabolism. Additionally, ophthalmologic and skin abnormalities are often present, suggesting that disturbed glutamate homeostasis affects tissues of ectodermal origin: brain, eye, and skin. Reporting glutamate and glutamine concentrations in patients with inborn errors of glutamate metabolism would provide additional insight into the pathophysiology.

## 1 INTRODUCTION

The amino acids glutamate and glutamine are key players in metabolism, functioning as both substrate and product in various enzymatic reactions.^1^ Glutamate is the main excitatory neurotransmitter in the central nervous system and triggers responses implicated in neuronal migration and differentiation, synapse remodelling and axon myelination.^2^, ^3^ Additionally, glutamate is the precursor of glutathione and the tricarboxylic acid (TCA) cycle intermediates and therefore plays a role in redox state and energy metabolism respectively.^4^ It is involved in amino acid synthesis and catabolism and as such involved in many metabolic pathways. Glutamate is directly metabolised by approximately 25 enzymes.^1^ Together with glutamine, glutamate plays an important role in ammonia detoxification by capturing ammonia—forming glutamine—and providing the urea cycle with intermediates.^5^ Glutamine is needed for NAD^+^ production, an important metabolite for redox reactions and is also incorporated into proteins, and a source of purines and pyrimidines.^6^, ^7^, ^8^, ^9^

Excess of glutamate and glutamine is harmful to cells. Glutamate excess in the brain is associated with glutamate excitotoxicity, a cascade of events that eventually leads to oxidative stress, toxicity, and cell death.^10^, ^11^ Glutamine excess leads to insufficient osmoregulation and subsequent cerebral edema.^6^, ^12^ Glutamate and glutamine concentrations are therefore strictly regulated.^13^ Defects in glutamate metabolism lead to metabolic diseases, as illustrated in this review.

Recently, a nosology for disorders of IEM has been proposed, of which the subcategory “disorders of glutamate metabolism” include glutamate transporters and receptors. Only one enzymatic defect was classified within this subcategory.^14^ We performed a literature review of defects in enzymes that directly metabolise glutamate caused by genetic errors, as these provide insight into the contributing role of deviant glutamine and glutamate levels to the pathophysiology. Seventeen inborn errors of glutamate metabolising enzymes have been reported, most of which are ultra‐rare. These were further categorised into defects disturbing the inter‐conversion of glutamine and glutamate; amino acid metabolism including those coupled to glutamate and α‐ketoglutarate transamination reactions; ammonia detoxification including urea cycle defects and other defects resulting in hyperammonemia; and glutathione metabolism including defects of the γ‐glutamyl‐cycle. We provide an overview of these inborn errors in an effort to ease the recognition.

### 1.1 Disorders of glutamine‐glutamate inter‐conversion

**Figure 1:**
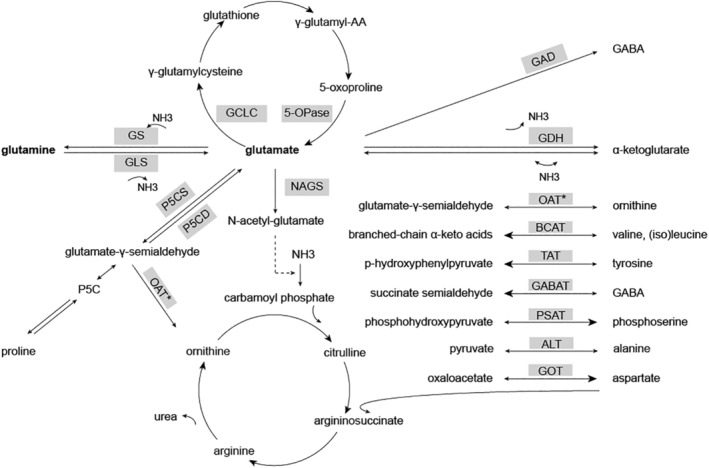
Metabolic pathways of glutamate metabolism in which genetic defects lead to metabolic disease. Clockwise from the top: γ‐glutamyl cycle, GABA synthesis, α‐ketoglutarate synthesis, α‐ketoglutarate/glutamate transamination with concomitant production or catabolism of amino acids and glutamate, urea cycle, proline synthesis and catabolism, glutamine synthesis, and catabolism. All enzymes are marked grey. 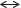 bidirectional reaction with preferred direction in bold; 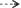 co‐factor of reaction. * OAT enzyme depicted twice

Glutamate and glutamine are interconverted by two enzymes (Figure 1). Glutamine synthetase (GS) catalyses the conversion of glutamate and ammonia into glutamine. It is ubiquitously expressed and is the only enzyme for glutamine synthesis.^15^, ^16^ The reverse reaction is catalysed by glutaminase (GLS) which hydrolyses glutamine to yield glutamate and ammonia. GLS exists in two isoforms: GLS, mainly expressed in kidney and brain, and GLS2, primarily expressed in liver.^17^, ^18^ Genetic defects in both GS and GLS have been reported (Table 1).

**Table 1:** Clinical and laboratory findings of inborn errors of glutamate metabolism based on the Human Phenotype Ontology (HPO) database and original articles

Disorder (OMIM) enzyme; *gene*; EC code	Number of described cases if <10; inheritance pattern	Clinical features	Biochemical diagnostics	Ref
**Disorders glutamine‐glutamate inter‐conversion**				
**Glutaminase hyperactivity** GLS; *GLS*; EC 3.5.1.2	n = 1 de novo, dominant	Development: Profound developmental delay Neurologic: axial hypotonia, kyphosis, agitated, and self‐injuring behaviour. Structural: white matter involvement Ophthalmologic: Neonatal bilateral cataracts	Plasma: mild hyperammonemia Urine: ↑ glutamate, ↓ glutamine CSF: =glutamate, =glutamine MRS brain: ↑ glutamate, ↓ glutamine, ↓ N‐acetylaspartate, ↑alanine, ↑ lactate	19
**Glutaminase loss‐of‐function** GLS; *GLS*; EC 3.5.1.2	n = 9 bi‐allelic, AR	Development: neonatal death (4/9), regression around age 7 years (2/9) dysarthria (5/9) Neurologic: status epilepticus with suppression burst patterns (4/9), truncal ataxia (7/9), hypertonia of limbs (4/9) Structural: white matter involvement, simplified gyral pattern, vasogenic cerebral oedema with subsequent gliosis and destruction (4/9), cerebellar atrophy with normal white matter (5/9) Opthalmologic: optic bilateral atrophy (2/9) Respiratory: Cheyne‐Stokes respiration	Dried bloodspot: ↑ glutamine, = glutamate	22, 24, 25
**Glutamine** **deficiency** (610015) GS; *GLUL*; EC 6.3.1.2	n = 4 bi‐allelic, AR	Development: severe global developmental delay (2/4), neonatal multi‐organ failure (2/4), death (2/4 neonatal; 1/4 age 6 years) Neurologic: seizures within first months of life (4/4), hypotonia (2/4), hyperreflexia (1/4) Structural: immature/hypomyelination (3/4), atrophy (3/4), abnormal gyration, hypoplasia corpus callosum (2/4) ventriculomegaly (1/4) sub‐ependymal/periventricular cysts (2/4) Dysmorphic features: broad/flat nasal root (2/4), low set ears (2/4), short limbs, flexion contractures, camptodactyly, ulnar deviation of the hands (1/4) Dermatologic: necrolytic erythema (2/4) Respiratory: apneu, decompensation (1/4)	Plasma: hyperammonemia, Borderline ↓ glutamine, = glutamate Urine: ↓ glutamine, = glutamate CSF: ↓ glutamine, = glutamate MRS: ↓ glutamine, =/↓ glutamate	26, 28
**Disorders of amino acid metabolism**				
**Hyperprolinemia II** (239510) P5CD; *ALDH4A1*; EC 1.2.1.88	Bi‐allelic, AR	Development: Intellectual disability Neurologic: Seizures	Plasma: ↑ proline, ↑ hydroxyproline, ↑ glycine (lactic acidosis excluded) ↓ PLP Urine: ↑ proline, ↑ hydroxyproline, ↑ P5C Fibroblasts: ↓ Enzyme activity	33, 34, 35, 95, 96
**Hereditary tyrosinaemia II** (276600) TAT; *TAT*; EC 2.6.1.5	Bi‐allelic, AR	Development: Intellectual disability, growth delay Neurological: seizures, microcephaly, self‐injury and other behavioural abnormalities, ataxia, tremor, neurological speech impairment Ophthalmologic: bilateral ocular lesions, inflammation conjunctivae, corneal ulcerations, photophobia Dermatologic: skin lesions	Plasma: ↑ tyrosine (>1200 μM, otherwise HTIII) ↑ p‐hydroxyphenylpyruvate, ↑ phenolic acids, = acids phenylalanine, methionine CSF: ↑ tyrosine Urine: ↑ phenolic acids Liver biopsy: ↓ enzymatic activity	37, 38, 97
**GABA transaminase deficiency** (613163) GABAT; *ABAT*; EC 2.6.1.19	Bi‐allelic, AR	Development: global developmental delay, death age < 10 years. Neurological: encephalopathy, seizures, lethargy, hyperreflexia, muscular hypotonia, high pitched cry, Structural: leukodystrophy, agenesis corpus callosum, cerebral atrophy, cerebellar hypoplasia and cysts, posterior fossa cyst Dysmorphic features: retrognathia, down‐slanted palpebral fissures, tall stature	CSF: ↑ GABA MRS: ↑ GABA, ↓ glutamine‐glutamate semi‐oval region Serum: ↑ growth hormone	40, 41, 98, 99
**Cerebral palsy, spastic quadriplegic, 1** (605363) GAD; *GAD1*; EC 4.1.1.15	n = 6 bi‐allelic, AR	Development: global developmental delay Neurological: seizures, hyperreflexia, cerebral palsy, Babinski sign	Not available	42
**Hypervalinemia and hyperleucine‐isoleucinemia** (238340) BCAT2; *BCAT2*; EC 2.6.1.42	n = 3 bi‐allelic, AR	Development: normal—delay Neurological: paroxysm occipital headache, mild memory decline, seizures Structural: white matter involvement Opthalmologic: retinal degeneration	Plasma: ↑ valine, ↑ isoleucine, ↑ leucine Urine: = branched‐chain α‐keto acids (excluding MSUD) MRS brain: ↓ n‐acetylaspartate	43, 44, 46, 47
**Mental retardation 49** (616281) ALT2; *GPT2*; 138 210	Bi‐allelic, AR	Development: Global developmental delay, failure to thrive Neurological: seizures, postnatal microcephaly, hypotonia with progressive spastic di/paraplegia with hyperreflexia, dysarthria Structural: subcortical hypomyelination, hypoplasia corpus callosum	Plasma: ↓ alanine, = glutamate, = glutamine Urine: = pyruvate, = lactate CSF: = glutamate, = glutamine, =/↓ alanine, = pyruvate, = lactate	49, 50, 100
**Neu‐laxova syndrome 2/phosphoserine aminotransferase deficiency** (616038/610992) PSAT; *PSAT1*; EC 2.6.1.52	Bi‐allelic, AR	Development: intrauterine growth retardation, decreased foetal movement, neonatal death Dysmorphic features: depressed nasal ridge, low set ears, abnormal pinna, short neck, high/cleft palate, micrognathia, sloping forehead, hypertelorism, acquired microcephaly, scoliosis, limb malformations, Dermatologic: ichthyosis, hyperkeratosis, oedema of acra.	Plasma: ↓ serine ↓ glycine CSF: ↓ serine ↓ glycine	53, 54, 55, 101
**Disorders of ammonia detoxification**				
**N‐acetylglutamate synthase deficiency** (237310) NAGS; *NAGS*; EC 2.3.1.1	Bi‐allelic, AR	Development: Developmental delay, failure to thrive, neonatal death Neurologic: Seizures, hypotonia, coma, aggressive behaviour, lethargy, vomiting Respiratory: respiratory distress	Plasma: hyperammonemia, ↓ citrulline, ↑ alanine, ↑ glutamine, ↑ glutamate (1 patient), Liver biopsy: ↓ enzyme activity	60, 102, 103
**Hyperinsulinemia‐hyperammonemia/hypoglycaemia 6** (606762) GDH; *GLUD*; EC 1.4.1.2	de novo/AD	Development: Intellectual disability Neurologic: hypoglycemic generalised absence‐type epileptic seizures, hypoglycemic coma, generalised dystonia	Plasma: hypoglycemia (provoked at protein intake), hyperammonemia (constant), = glutamine CSF: GABA normal (only measured in four patients) Lymphoblasts: ↑ GDH activity by reduced sensitivity to GTP	64, 104, 105
**Glutamic‐Oxaloacetic Transaminase 2 deficiency** AST/GOT2; *GOT2*; EC 2.6.1.1	n = 2 bi‐allelic, AR	Development: Developmental delay Neurologic: clonic seizures upper limbs, acquired microcephaly, hypotonia	Plasma: ↓ serine, =/↑ glycine, ↑ citrulline, ↑ lactate, hyperammonemia, = glutamine, = glutamate	65
**Cutis laxa 3A/3** (219 150/616603) **Spastic paraplegia 9A/B (adult phenotype)** (601 162/616586) P5CS; *ALDH18A1*; EC 2.7.2.41	bi‐ mono‐allelic, AR/AD/ de novo	Development: Developmental delay, pre‐postnatal growth retardation Neurologic: neurodegeneration, hypotonia, microcephaly, movement disorder Structural: hypomyelination, cortical atrophy and hypoplastic corpus callosum, tortuosity of brain vessels, cerebellar abnormalities Dermatologic (only cutis laxa): thin wrinkled skin with visible veins. Ophthalmologic: neonatal cataract, corneal clouding, nystagmus	Plasma: hyperammonemia, ↓ proline, ↓ornithine, ↓citrulline (SP), ↓ arginine, = glutamine, = alanine MRS: ↓ creatine SP specific: ↓ citrulline, low sum of involved amino acids	67, 68, 70, 72
**Gyrate atrophy choroid & retina** (258870) OAT; *OAT*; EC 2.6.1.13	Bi‐allelic, AR	Development: failure to thrive Neurologic: proximal muscle weakness Ophthalmologic: myopia, nyctalopia, infantile cataracts, progressive chorioretinal atrophy, adult blindness	Plasma: ↑ ornithine, ↓ creatine Urine: ↓ creatine, ↑ ornithine, ↑ lysine ↑ arginine Muscle biopsy: ↓ creatine, type II muscle fibre atrophy with tubular aggregates. Fibroblasts, leukoblasts: ↓ OAT activity Neonatal: reversed enzyme direction ➔ ↓ citrulline, ↑ proline, ↓ ornithine, ↓ arginine, hyperammonemia, ↑ glutamine. Diagnosis: ↑ ratio proline/citrulline	73, 74, 75
**Disorders of glutathione metabolism**				
**γ‐glutamyl‐cysteine synthetase deficiency** (230450) GCLC; *GCLC*; EC: 6.3.2.2	Bi‐allelic, AR	Development: Developmental delay Neurologic: late onset spinocerebellar degeneration, peripheral neuropathy, myopathy. Haematologic: infantile onset haemolytic anaemia Gastro‐intestinal: hepatosplenomegaly with transient jaundice	Erythrocytes: ↓ glutathione (<5%) ↓ y‐glutamyl‐cysteine Erythrocytes, leukocytes, fibroblasts: ↓GCLC activity (<10%) Urine: ↑ 5‐oxoproline (excluding GSS deficiency)	77, 79, 80, 81
**5‐oxoprolinase deficiency** (260005) 5‐OPase; *OPLAH*; EC: 3.5.2.9	Bi‐allelic, AR/AD	Unaffected/Inconsistent Gastro‐intestinal: enterocolitis, diarrhoea, vomiting, abdominal pain Nephrologic: nephrolithiasis	Urine: ↑ 5‐oxoproline Erythrocytes: = glutathione (excluding GSS deficiency)	82, 83, 84, 85

*Note*: =, normal levels; ↓, decreased levels; ↑, increased levels. Abbreviations: AR, autosomal recessive; AD, autosomal dominant; CSF, cerebrospinal fluid; GLS, glutaminase; GS, glutamine synthetase; GLUL, glutamate‐ammonia ligase; P5CD, pyrroline‐5‐carboxylate dehydrogenase; ALDH, aldehyde dehydrogenase; TAT, tyrosine aminotransferase; GABAT, gamma‐aminobutyric acid transferase; GAD1, glutamate decarboxylase; BCAT, branched‐chain amino acid aminotransferase; ALT, alanine aminotransferase; GPT, glutamate pyruvate transferase; PSAT, phosphoserine transaminase; NAGS, N‐acetylglutamate synthase; GDH, GLUD glutamate dehydrogenase; AST, aspartate transaminase; GOT, glutamic oxaloacetic transaminase; P5CS, pyrroline‐5‐carboxylate synthetase; OAT, ornithine aminotransferase; GCLC, Glutamate‐Cysteine Ligase Catalytic Subunit; 5‐OPase, 5‐oxoprolinase.

A de novo hypermorphic *GLS* variant has recently been reported in a single patient affected with cataract, profound developmental delay, axial hypotonia and agitated, self‐injuring behaviour.^19^ Glutamate and glutamine concentrations in plasma and CSF from the patient were within the normal range. In urine and in brain, as detected by in vivo magnetic resonance spectroscopic imaging (MRSI), glutamate concentrations were increased and glutamine concentrations decreased, in line with the tissue‐specific expression of GLS.^17^ Glutamate excess is thought to underlie the pathophysiology of the disease by reducing the scavenging capacity for oxygen radicals and making the cell more susceptive for oxidative stress, which is associated with cataract, neurodegenerative disorders and agitated, self‐injuring behaviour.^10^, ^20^, ^21^

*GLS* loss‐of‐function variants have been reported recently in unrelated families. In two families, patients clinically presented with neonatal respiratory dysfunction and fatal, refractory status epilepticus.^22^ MRI of the brain showed extensive cerebral oedema and white matter involvement. The *GLS* variants were discovered by post‐mortem exome sequencing. Retrospective amino acid analyses of stored dried bloodspots from neonatal screening revealed increased glutamine values. Increased glutamine is a possible cause of the on‐going epileptic activity secondary to cerebral edema.^12^, ^23^ Glutamate values were unaltered in bloodspots. Considering the importance of glutamate in signal transduction and myelin synthesis in the brain, decreased glutamate levels might also have played a contributing pathogenic role in this metabolic disease by causing epilepsy and secondary cerebral oedema. Glutamate levels in the brain could however not be determined. In another family, decreased GLS expression, as a consequence of a homozygous copy number variant in *GLS*, was detected in two brothers who initially developed normally, but from the age of 7 years developed progressive optic atrophy, truncal ataxia and hypertonia in the limbs. MRI of the brain showed cerebellar atrophy with normal white matter. Glutamine and glutamate values were not reported.^24^ Three other unrelated patients with GLS deficiency, as a consequence of tandem repeat expansion in GLS, presented with early‐onset delay in overall development, progressive ataxia and elevated glutamine plasma levels. Glutamate plasma levels were unaltered.^25^

Congenital glutamine synthetase deficiency, caused by bi‐allelic mutations is the *GLUL* gene, has been described in four unrelated children. All the patients suffered from seizures which developed either after birth or within few months of life. Two suffered from necrolytic migratory erythema and died within the first month of life from multi‐organ failure.^26^ These exhibited dysmorphic features including a broad, flat nasal root and low set ears. The third patient developed drug‐resistant tonic‐clonic seizures and died at 6 years of age from acute respiratory decompensation. The fourth patient was recently described and mainly suffers from seizures. Glutamine concentrations in these patients were either strongly or borderline decreased in plasma, urine, CSF and in brain (as detected by MRS), in line with the ubiquitous expression of GS. Plasma ammonia levels were increased. Glutamine supplementation in the third patient normalised glutamine concentrations in plasma, improved EEG and even reduced seizure attacks.^27^ In the fourth patient, glutamine supplementation also normalised glutamine levels and decreased ammonia levels, however follow‐up is still performed to study the clinical effect.^28^ Hyperammonemia and NAD^+^ deficiency secondary to glutamine deficiency are likely the pathogenic factors causing the phenotype. Hyperammonemia is highly toxic for the brain and associated with encephalopathy and seizures, and NAD^+^ is important for numerous redox reactions in the cell.^6^, ^29^ Recently, a new biological function of GS for motility and migration of endothelial cells has been revealed, which contributes to the formation of new vessels in development and disease.^30^ Whether this plays a contributing role to the clinical phenotype of GS deficiency has not been elucidated yet.

In conclusion, defects in enzymes interconverting glutamate and glutamine lead to different, mainly neurological, phenotypes. They result in disturbed levels of glutamate and glutamine in several body fluids and tissues which plays an important pathophysiologic role.

### 1.2 Disorders of amino acid metabolism

Glutamate functions as a substrate for amino acid synthesis and as a product of amino acid catabolism (Figure 1). Defects in these enzymes of amino acid metabolism, including several transaminases, lead to deviant amino acid levels and to a broad spectrum of clinical phenotypes, depending on the amino acid function (Table 1). According to the most recent proposed nosology for IEMs, these can be subdivided by their involved amino acids.^14^ We decided to group them in this review, as these all involve glutamate metabolism.

P5C dehydrogenase (P5CD) degrades proline via Δ‐1‐pyrroline‐5‐carboxylate (P5C) into glutamate.^31^ Deficiency of P5CD results in hyperprolinemia with concomitant increase of urinary P5C (hyperprolinemia type II; HPII).^32^, ^33^ These high P5C levels deactivate PLP (vitamin B6), a co‐factor for many enzymes.^34^, ^35^ An effect of P5CD deficiency on glutamate levels has not been reported. This disorder presents with intellectual disability and fever‐provoked PLP‐responsive epileptic seizures ascribed to PLP deficiency, which is a common cause of epilepsy.^36^ Patients may however also remain free of symptoms.

The reverse reaction, the formation of proline from glutamate, is catalysed by P5C synthetase (P5CS). P5CS deficiency is further described under “Disorders of urea‐cycle and ammonia metabolism.”

Tyrosine aminotransferase (TAT) transaminates tyrosine, forming p‐hydroxyphenylpyruvate and glutamate. TAT deficiency leads to increased levels of tyrosine in plasma and CSF together with increased levels of its catabolic metabolites in plasma and urine.^37^, ^38^ Glutamate levels have not been reported in this disease, suggesting that they were not significantly altered. Deposition of tyrosine crystals leads to eye and skin lesions in patients with TAT deficiency, resulting in infancy‐onset ophthalmological and dermatological symptoms. The phenotype is further characterised by seizures and self‐injuring and difficult behaviour.

GABA transaminase (GABAT) converts GABA into succinate semialdehyde and glutamate. Patients with GABAT deficiency show high GABA levels in CSF and on brain MRS and have increased growth hormone release induced by GABA. These children suffer from a severe neurological phenotype including seizures, hypersomnolence and choreoathetosis, and accelerated growth. Symptoms are likely due to increased GABA levels, detected in plasma, CSF, and basal ganglia.^39^ Although no contributed role of potentially altered glutamate levels to the phenotype has been described, a disturbed GABA‐glutamate ratio is postulated to contribute to this metabolic disorder.^40^, ^41^ Mutations in the *GAD1* gene, encoding glutamate decarboxylase which irreversibly decarboxylates glutamate into GABA, have been described in one family with inherited spastic quadriplegic cerebral palsy type 1, seizures and developmental delay.^42^

Branched‐chain amino acid transferase 2 (BCAT2) deficiency has been described in a single patient who presented with high plasma levels of the branched‐chain amino acids leucine, isoleucine and valine.^43^ The patient suffers from paroxysmal occipital headache, mild memory decline and retinal degeneration. A brother and sister with leucine‐isoleucine abnormalities presented with seizures, developmental delay, retinal degeneration, and died at the age of 3 years. A *BCAT2* mutation was assumed but not validated.^44^ Recently, a genome wide association study showed a clear association between *BCAT2* missense mutations and increased valine levels, however no phenotypes were described.^45^ It is hypothesised that BCAT provides nitrogen for optimal glutamate synthesis—which is supported by the finding that BCAT2 inhibition decreases glutamate levels—and that the clinical phenotype of BCAT2 deficiency is attributed to disturbed glutamate synthesis rather than the increase in branched‐chain amino acids.^46^, ^47^, ^48^ However, thus far glutamate levels were not reported in BCAT2 deficient patients, precluding definite conclusions.

Alanine transaminase 2 (ALT2) deficiency leads to low concentrations of alanine in plasma and in some cases also in CSF.^49^, ^50^ Affected patients clinically present with developmental delay, spastic paraplegia and sporadically with seizures. It has been postulated that ALT2 deficiency leads to decreased glutamate production in the brain disturbing the glutamate‐glutamine/lactate‐alanine shuttle and synaptic neurotransmitter release, which might contribute to the pathogenesis.^50^ The strictly neurological effect of ALT2 deficiency can be explained by the neuronal expression of ALT2 and possible compensation by *ALT1* expressed in other tissues. Although glutamate levels were normal in CSF and plasma, this does not exclude the possibility of deviant levels in the brain and a local effect. There is contradictory data on the preferable direction of ALT2.^51^, ^52^ The decreased alanine concentrations in ALT2 deficiency however suggests that the preferable direction of ALT2 is towards alanine synthesis.

Phosphoserine aminotransferase (PSAT) uses the amino‐group of glutamate for serine synthesis.^53^ PSAT deficiency has been reported in patients with Neu‐Laxova syndrome 2, characterised by central nervous system anomalies, facial dysmorphic features, anomalies of limb and genitalia, intrauterine growth retardation, skin disorders, and other congenital abnormalities.^54^ An overlapping phenotype caused by PSAT mutations is described in one family under the name phosphoserine aminotransferase deficiency.^55^ It is questionable whether this concerns the same syndrome. Neu‐Laxova syndrome can also be caused by other genetic defects interfering the serine biosynthesis pathway. Serine is a precursor of important metabolites such as nucleotides, phospholipids and neurotransmitters. Therefore, the pathophysiology in PSAT deficiency and in other serine deficiency disorders with similar symptoms is ascribed to the lack of serine.^56^ A potential contributing role of glutamate has not been described.

In conclusion, defects of P5C dehydrogenase and transaminases producing or metabolising amino acids with concomitant glutamate conversion lead to different metabolic profiles. Glutamate and glutamine levels are not consistently reported, precluding conclusions about their contributing role to the pathophysiology underlying the broad spectrum of clinical phenotypes. The pathophysiology of these disorders is postulated to be due to disturbed metabolic pathways downstream from the particular affected enzyme. Nevertheless, tissue specific abnormalities and pathophysiologic involvement of glutamate and glutamine may still play a role in these inborn errors of glutamate metabolism.

### 1.3 Disorders of ammonia detoxification

The urea cycle is initiated by N‐acetyl glutamate synthase (NAGS), which catalyses the conversion of glutamate into N‐acetyl glutamate (NAG), an obligatory co‐factor of the first enzyme of the urea cycle, carbamoyl phosphate synthetase 1 (CPS1).^57^ Primary urea‐cycle disorders (UCDs) caused by defects in urea cycle enzymes, including NAGS deficiency, are characterised by altered levels of citrulline, ornithine, and arginine, depending on the site of the metabolic block. This hampered urea cycle leads to hyperammonemia, which can be detoxified by the formation of glutamine and alanine (non‐toxic ammonia carriers) from glutamate and pyruvate respectively. Toxic hyperammonemia results in the typical clinical phenotype characterised by headaches, lethargy, and seizures.^58^ NAGS deficiency leads to inactive CPS1 and a hampered urea cycle resulting in hyperammonemia, increased glutamine concentrations and hypocitrullinemia. This leads to the typical clinical presentation of UCDs.^59^ NAGSD theoretically leads to glutamate excess, which was indeed reported in one patient but remained unreported in other patients.^60^ In addition to primary UCDs, four other defects in glutamate metabolism disturbing the urea cycle and ammonia metabolism have been described (Table 1).

Glutamate dehydrogenase (GDH) catalyses the deamination of glutamate into α‐ketoglutarate and free ammonia.^61^ Patients with *GDH* gain‐of‐function mutations—resulting in increased sensitivity to ADP allosteric activation—present with neonatal‐infantile onset hyperinsulinism‐hyperammonemia (HI‐HA syndrome), also called hyperinsulinism hypoglycaemic 6 syndrome.^62^, ^63^ Hyperammonemia is constant and postulated to result either from increased glutamate deamination directly leading to high ammonia or from glutamate deficiency leading to reduced NAG synthesis, although the levels of urea cycle intermediates and of glutamine remain normal. In parallel, increased GDH activity results in a fuelled pancreatic TCA cycle through α‐ketoglutarate resulting in insulin secretion and hypoglycemia. Patients suffer from epileptic seizures—amenable to diazoxide treatment—and developmental delay, but typical symptoms of hyperammonemia like headaches and encephalopathy are absent.^64^ As epileptic seizures occur independently of glucose concentrations, epileptic seizures are likely cause by hyperammonemia or a disequilibrium of glutamate:GABA.^64^ *GDH* mutations follow an autosomal dominant trait. However, mutations in the sulfonylurea receptor, either recessive or dominant, causing HIHA are also described.

Glutamic oxaloacetic transaminase 2 (GOT2) transfers the amino‐group of glutamate to produce aspartate. GOT2 deficiency was recently described in patients who presented with mild hypercitrullinemia, mild hyperammonemia and normal glutamine, arginine and ornithine levels.^65^ Additionally, affected patients had secondary serine deficiency as a consequence of a hampered aspartate‐malate cycle and suffered from epileptic seizures and acquired microcephaly ascribed to the secondary serine synthesis defect. This defect can be cross‐categorised in disorders of amino acid metabolism.

Ornithine, substrate in the urea cycle, is synthesised by two enzymes directly metabolising glutamate. Δ‐1‐pyrroline‐5‐carboxylate synthetase (P5CS) converts glutamate into glutamate‐γ‐semialdehyde. This is in equilibrium with P5C and can be converted into ornithine. P5CS deficiency results in low ornithine and subsequent citrulline and arginine levels with mild fasting hyperammonemia. Despite the hampered urea cycle, glutamine concentrations are not increased in these patients. This defect additionally results in low proline levels, the product of P5C. Patients present with symptoms ranging from severe cutis laxa to adult‐onset spastic paraplegia (depending on the presence of mono‐ or bi‐allelic mutations) with concomitant hypermobility of the joints, neurodegeneration, and bilateral cataracts or corneal clouding.^66^, ^67^, ^68^, ^69^, ^70^, ^71^ These symptoms are in line with the role of proline in collagen and elastin synthesis, protein synthesis, oxidative stress defence in addition to a possible role as an inhibitory neurotransmitter.^72^

Ornithine aminotransferase (OAT) converts glutamate‐γ‐semialdehyde into ornithine coupled to glutamate transamination. In the neonatal period, the enzyme is ornithine‐producing. Neonatal‐onset OAT deficiency leads to low ornithine, hyperammonemia, high glutamine concentrations, and failure to thrive.^73^, ^74^ In infancy and adulthood, the enzyme is ornithine‐catabolising and its deficiency results in increased ornithine levels and concomitant inhibition of creatine synthesis. Patients present mainly with gyrate atrophy of the choroid and retina and other ophthalmological abnormalities, although some have also been described with developmental delay. These symptoms are postulated to result from either creatine deficiency or toxicity of ornithine and its downstream metabolites spermine and spermidine, which induce retinal cell death and photoreceptor degeneration via oxidative stress.^75^, ^76^

Taken together, all inborn errors of glutamate metabolism leading to defects of the urea‐cycle or ammonia metabolism show hyperammonemia. However, in GOT2 deficiency, P5CS deficiency and GDH hyperactivity hyperammonemia is mild and does not result in glutamine excess. The pathophysiology of the primary UCD's is assigned to hyperammonemia, while the pathophysiology of the other disorders of ammonia metabolism are postulated to be due to disturbed metabolic pathways downstream from the particular affected enzyme.

### 1.4 Disorders of glutathione metabolism

Glutamate is one of the intermediaries of the γ‐glutamyl‐cycle, involving synthesis and degradation of the important anti‐oxidant glutathione. Two of the disorders of the γ‐glutamyl‐cycle disorders are directly involved in glutamate metabolism (Figure 1, Table 1).

γ‐Glutamyl‐cysteine synthetase (GCLC) catalyses the conjugation of glutamate and cysteine to γ‐glutamyl‐cysteine and is the first and rate‐limiting step of glutathione biosynthesis.^77^ GCLC deficiency leads to extremely low levels of erythrocytic glutathione and γ‐glutamyl‐cysteine which result in infantile‐adult onset haemolytic anaemia.^78^ Other reported features are neurological problems, amino aciduria, reticulocytosis, and hepatosplenomegaly with transient jaundice but whether these are related to the enzyme defect remains unclear.^79^, ^80^, ^81^

5‐Oxoprolinase (5‐OPase) catalyses the last degradation step of glutathione into glutamate by the hydrolysis of 5‐oxo‐proline.^82^ 5‐OPase deficiency (OPLAHD) leads to 5‐oxoprolinuria, normal erythrocytic glutathione levels and an inconsistent—or even no—clinical presentation including gastro‐intestinal features and hypoglycemia.^83^, ^84^ The lack of a consistent clinical picture and the normal glutathione levels lead to the hypothesis that 5‐OPase deficiency is a benign disorder, although a pathogenic role of 5‐oxoprolinase deficiency remains possible as specific clinical features may only become obvious later in life.^84^, ^85^

In these γ‐glutamyl‐cycle disorders, glutamate and glutamine levels have not been reported. The pathophysiology is likely predominantly based on oxidative stress, as this cycle plays an important role in metabolism of glutathione.

### 1.5 Drawing parallels by comparison of phenotypic abnormalities from Human Phenotype Ontology (HPO)

In order to create insight into the clinical effect of disturbed glutamate metabolism, we explored clinical parallels between the disorders of glutamate metabolism based on a hierarchical database. This database was created based on all phenotypic abnormalities and their correlated superclasses as reported in the HPO database (Table S1). For the recently described GLS hyperactivity, GLS loss‐of‐function and GOT2 deficiency, this information was obtained from the original articles as these have not yet been included in HPO. We obtained a hierarchical database of 539 phenotypic abnormalities including the correlated superclasses. To analyse whether disturbed glutamate metabolism leads to specific clinical consequences, the prevalence of phenotypic abnormalities within the 17 inborn errors of metabolism was plotted against their prevalence within all 10.204 Mendelian disorders listed in HPO. A prevalence rate > 2 was used. The following phenotypic abnormalities were excluded from the selection: metabolic abnormalities other than glutamate and glutamine (as we focused on clinical phenotypes rather than metabolic phenotypes); duplicates; upper superclasses (as these are non‐specific abnormalities); and phenotypic abnormalities that only presented in one of the 17 inborn errors. The 74 phenotypic abnormalities that were > 2 times more prevalent within inborn errors of glutamate metabolism than in all Mendelian disorders were used for further analyses. Of those, the prevalence rate of remarkable phenotypic abnormalities was also compared with the prevalence rate of all HPO disorders with metabolic abnormalities (used as a measure for inborn errors of metabolism).

Perhaps not surprisingly, the phenotypic abnormalities that are remarkably more prevalent within inborn errors of glutamate metabolism than in all Mendelian disorders and in the subgroup of all mendelian disorders with metabolic abnormalities, are abnormalities of the nervous system. All inborn errors of glutamate metabolism—except for OAT and OPLAH deficiency—result in neurodevelopmental abnormalities, of which GLS hyperactivity and loss, GS, GABAT, ALT2, PSAT, GAD, GOT2, and P5CS deficiency present in a more severe, global developmental delay. This is 14× more prevalent than in all Mendelian disorders and 8.9× more prevalent than in all disorders with metabolic abnormalities. Additionally, seizures are more common in patients with defects of GLS, GS, P5CD, GABAT, BCAT2, ALT, PSAT, NASGS, GAD, GOT2 and in patients with HIHA (3.9× compared to all Mendelian disorders and 2.5× compared to all disorders with metabolic abnormalities). White matter and myelination abnormalities are frequently reported in these patients. Coma (12.8×/4.0), lethargy (9.8×/3.5) and vomiting (9.7×/3.7) are also more common phenotypic abnormalities (compared to all Mendelian disorders/disorders with metabolic abnormalities), especially—but not only—in disorders of urea cycle and ammonia metabolism. Abnormalities of the lens and skin are also more prevalent in patients with an inborn error of glutamate metabolism. Cataract occurs in patients with GLS hyperactivity and P5CS and OAT deficiencies and is 2.8× more common in these inborn errors compared to all Mendelian disorders and 2.2× compared to all disorders with metabolic abnormalities. Different skin abnormalities are seen in patients with GS deficiency, GLS hyperactivity, PSAT and TAT deficiency and are 2.5‐3.0× more common in glutamate related disorders compared to other Mendelian disorders, but only 1.3‐1.5× compared to other disorders with metabolic abnormalities (Table S1).

## 2 DISCUSSION

Inborn errors in enzymes of glutamate metabolism lead to distinct clinical and biochemical phenotypes, which can be classified in disorders affecting the inter‐conversion of glutamine and glutamate, amino acid metabolism, ammonia detoxification and glutathione metabolism. In this review, we provide an overview of the clinical and biochemical phenotype of these defects in an effort to create insight into their pathophysiology and the contributing role of deviant glutamate and glutamine concentrations and to ease the recognition of these ultra‐rare metabolic disorders. Inborn errors of glutamate metabolism caused by transporters and receptors are disregarded in this review.

Several families with GLS defects were recently described, presenting with overlapping but also diverging clinical and biochemical symptoms. We suspect that the ensuing biochemical and clinical picture is the reflection of residual GLS activity. Awareness of this phenotypic spectrum is needed for recognition and diagnosis of these patients.^22^, ^24^, ^25^

Glutamate and glutamine concentrations are most aberrant in defects of the two enzymes directly interconverting these amino acids: GLS and GS. Interestingly, in GLS hyperactivity, glutamate and glutamine levels are normal in plasma and CSF, while both are deviant when analysed using brain MRS and in urine in line with tissue specific expression of GLS.^19^ The discrepancy between CSF and brain MRS might be explained by the degree to which glutamate and glutamine levels are controlled by GLS. CSF is formed out of plasma by the plexus choroid, which consists of glial cells in which glutamate and glutamine levels are regulated by GS rather than GLS.^86^, ^87^ This illustrates that normal CSF values do not exclude the possibility of abnormal glutamate values in the brain. In patients with GLS or GS loss‐of‐function, glutamine concentrations were deviant in bloodspots or plasma, while glutamate was normal, possibly due to dietary intake and other glutamate metabolising enzymes. Local glutamate deficiency in the brain may, however, contribute to the epileptic phenotype in GLS loss‐of‐function, given the role of glutamate as the main neurotransmitter. Brain MRSI might therefore be indicated when a defect in glutamate metabolism is suspected. MRSI clinical field strength is a suitable approach for this. A higher magnetic field can even distinguish the closely positioned glutamine and glutamate peaks better and can even show regional differences of these metabolites.

This example illustrates that biochemical analysis has it pitfalls and that negative biochemical results should not exclude a diagnosis. The report of glutamate and glutamine values in additional patients with inborn errors of glutamate metabolism will create additional insight into the predictive value of these metabolites as biomarkers. Genetic diagnostics alone also has its pitfalls. The identification of disease causing variants might be hampered by the current paradigm that mono‐allelic variants in enzyme‐encoding genes are usually harmless. GLS hyperactivity illustrates that mono‐allelic, dominant enzyme defects can be harmful as well. Whole‐exome‐sequencing (WES), the preferred method to date, captures only a small portion of the genome and is limited in its capacity to detect intronic defects, copy number variants or regions with low coverage. Therefore, the combination of deep clinical and biochemical phenotyping with genetic analysis remains important, which should also be kept in mind when interpreting Table 1. GLS loss‐of‐function is a good example of this, as deep phenotyping and increased glutamine values in different patients pointed to GLS loss‐of‐function, despite the absence detected genetic alterations with WES. Further analysis with whole genome sequencing revealed repeat expansions in *GLS* as the underlying genetic cause.

Interestingly, both glutamine deficiency and glutamate excess may disturb the cellular redox status. In GS deficiency, glutamine deficiency results in reduced NAD^+^ synthesis, which plays an important role in redox reactions.^6^ In GLS hyperactivity, glutamate excess is postulated to decrease redox buffer capacity.^19^ As oxidative stress is associated with cataract, neurodegenerative disorders and epilepsy, this is a likely key player in the pathophysiology of these inborn errors of glutamate metabolism.^10^, ^20^, ^88^

In UCDs, glutamine concentrations are increased as a consequence of hyperammonemia. It is remarkable that both glutamine excess (in these UCDs) and glutamine deficiency (in GS deficiency) can be accompanied by hyperammonemia, which is likely to play a pathophysiological role in these inborn errors. In primary UCD's, glutamine concentrations are increased putatively as a consequence of hyperammonemia‐induced GS in an attempt to detoxify ammonia.^5^, ^89^ Conversely, glutamine concentrations remain normal despite hyperammonemia in GOT2 deficiency, P5CS deficiency and GDH hyperactivity. In these disorders, hyperammonemia is mild and might therefore not trigger excess glutamine formation.

In all other inborn errors of glutamate metabolism, glutamate concentrations have not been reported, therefore precluding conclusions. If these are presumed to be normal, this points to the regulation of glutamate levels by other enzymes of glutamate metabolism. The pathogenesis of these other errors is often ascribed to deficiency or toxic accumulation of substrate or product of the defective enzyme, as explicated in the main text above. However, as seen in GLS hyperactivity, normal concentrations of glutamate and glutamine in plasma and CSF do not exclude local alterations, which might contribute to the pathophysiology as well. Even more locally oriented, in the patient with GABAT deficiency no abnormalities were detected in plasma or CSF, but locally decreased glutamate concentrations in the semi‐oval region of the brain were detected on brain MRS and might possibly play a pathogenic role.^90^ Also, mildly decreased glutamine concentrations or mildly increased glutamate concentrations may have been detected but attributed to pre‐analytical conditions rather than a reflection of in vivo metabolism as glutamine is easily spontaneously interconverted into glutamate. The study of glutamate and glutamine and their ratio in carefully collected body fluids—as performed in GS deficiency and GLS hyperactivity—may elucidate alterations of glutamate and glutamine levels in inborn errors of glutamate metabolism. Several in vivo techniques are available to study glutamate and glutamine separately, for an overview see Ramadan et al.^91^

Inborn errors of metabolism that have a related biochemical ground often have similar clinical features. Drawing phenotypic parallels of all 17 inborn errors (both HPO phenotypic subclasses and their correlated superclasses) provided interesting leads towards similar pathophysiology. Neurologically, developmental delay, seizures and hypotonia are more common in these inborn errors compared to the prevalence in Mendelian disorders in general and in disorders with metabolic abnormalities, in line with the role of glutamate as the main neurotransmitter (Table S1). It is however remarkable that the patient with GLS hyperactivity and glutamate excess in the brain did not develop seizures. Glutamate induces myelination of axons and it is therefore not surprising that white matter and myelin abnormalities are frequently reported in patients with an inborn error of glutamate metabolism.^2^ Although this is an a‐specific sign seen in many disorders, it is more prevalent in glutamate related disorders than in all Mendelian disorders (14‐16×) and in all disorders with metabolic abnormalities (9‐13×). Patients with inborn errors of glutamate metabolism caused by defects of glutamate receptors and transporters also present with neurological disorders, like seizures, ataxia and developmental delay, underlying the clinical importance of balanced glutamate metabolism for the human brain.^14^

Interestingly, ectodermal structures seem more affected in inborn errors of glutamate metabolism as both neurologic abnormalities, skin and lens abnormalities are seen. Other ophthalmological features that are seen in glutamate related disorders are optic atrophy in some GLS loss patients; herpetiform corneal ulcerations in patients with TAT deficiency; chorioretinal atrophy in OAT deficient patients; and retinal degeneration in BCAT2 deficient patients. Patients with glutamate receptor 6 deficiency, present with congenital night blindness.^14^ Interestingly, the brain, skin, lens, optic nerve, cornea, and retina are all of ectodermal origin and therefore share similarities in expression and regulation of glutamate receptors.^92^, ^93^, ^94^ This suggests that disturbed glutamate homeostasis might not only affect the brain, but also these other ectodermal structures.

Altogether, 17 defects of enzymes that directly metabolise glutamate have been described so far. Data on glutamate and glutamine concentrations in patients with inborn errors in enzymes of glutamate metabolism should be measured and collected as they provide additional insight into the contributing role of deviant concentrations of these amino acids in the pathophysiology.

## Supporting information


**Appendix S1**: Tables
